# Segmentation of compacted and non compacted left ventricular mass with a semi-automatic method

**DOI:** 10.1186/1532-429X-17-S1-P55

**Published:** 2015-02-03

**Authors:** Stéphanie Bricq, Julien Frandon, Daniel Fagret, Alexandre Cochet, Alexis Jacquier, Alain Lalande

**Affiliations:** 1 Le2i UMR CNRS 6306, Dijon, France; 2 Service d'IRM, CHU Dijon, Dijon, France; 3 Service de radiologie, CHU Grenoble, Grenoble, France; 4 Service de radiologie, CHU La Timone, Marseille, France; 5 Médecine Nucléaire, CHU Grenoble, Grenoble, France

## Background

The diagnostic of left ventricular (LV) non compaction is still a challenge in clinical practice. There is a lack of reference method able to automatically quantify the total amount of LV trabeculations. Many algorithms have been proposed to determine endocardium and epicardium borders ranging from semi-automated to fully automated methods. However most of the methods were not designed to take into account papillary muscles and trabeculae. Here we propose a global framework to detect non-compacted, endocardium and epicardium contours with minimal user interaction, taking into account papillary muscles and trabeculae. Preliminary results on the normal non-compacted mass obtained from 20 healthy volunteers are presented.

## Methods

Proposed method is a semi-automatic one. The user has to click in the LV cavity on one slice. Using this seed, the non-compacted contour which is a homogeneous area in term of intensity, is obtained using a region growing algorithm. This non-compacted contour is then used for the initialization of a variational B-spline level-set to determine the endocardium border. This border is used as initialization for a region growing algorithm to obtain the epicardial border. Papillary muscles and trabeculations are then segmented using a semi-automatic threshold tool. Finally, compacted (C) mass, non-compacted (NC) mass, end-diastolic volume (EDV) and NC/C ratio are computed.

Analysis was performed on MR examinations obtained from twenty healthy subjects (mean age 46 ± 17 years, 8 men). All subjects were completely asymptomatic with no known risk factors or history of cardiac disease. Segmentation was performed by two independent observers on the diastolic frame from contiguous short axis cine-MR images covering the LV. Inter-observer measurement reproducibility was assessed thanks to Bland-Altman analysis and to the correlation coefficient calculation between the different volumes obtained by the two observers.

## Results

**Figure 1:**
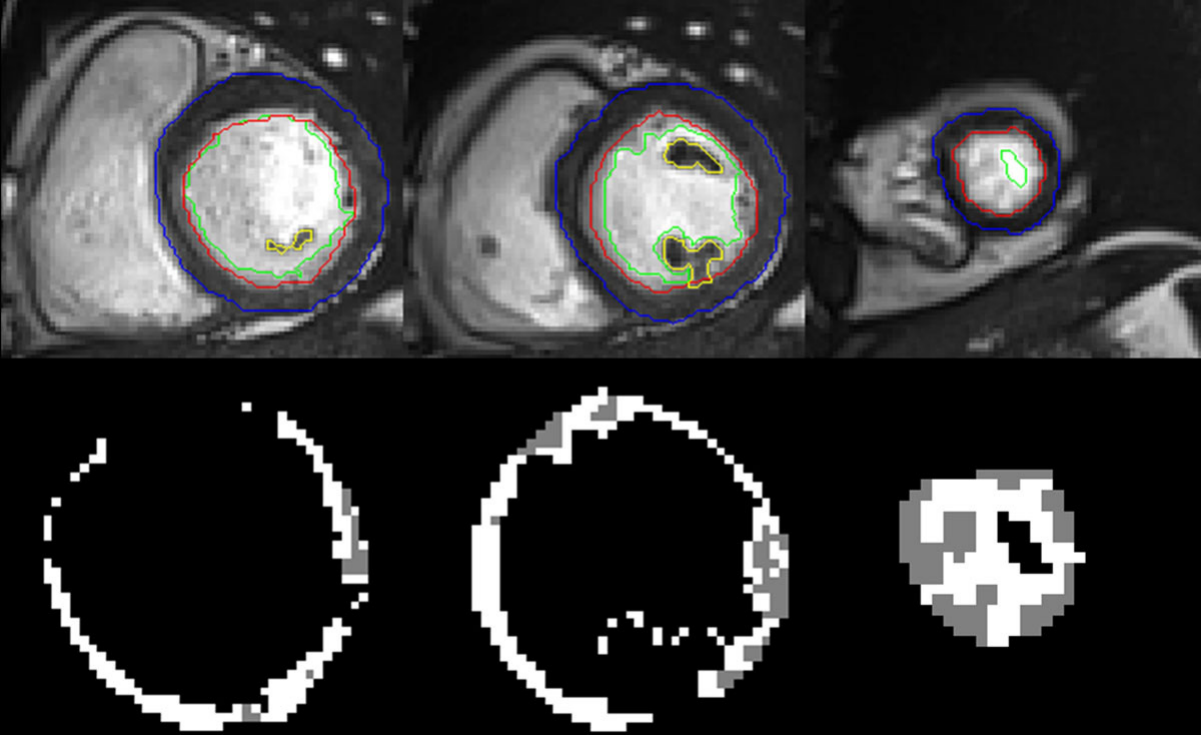
Example of the left ventricular segmentation from base to apex **(top)**: epicardial border in blue, endocardial border in red, trabeculae border in green. Papillary muscles were segmented and included into the compacted mass (yellow line). For the calculation of the non compacted mass **(bottom)**, trabeculae (grey) were segmented using a semi-automatic threshold tool to suppress blood (white).

Total processing time is less than 20 min for each patient. Figure 1 shows an example of the segmentation. Absolute values of all the parameters for normal subjects are presented in Table 1, as the inter-observer evaluation. The compacted mass is around 58 g/m2 and the non-compacted around 7 g/m2. Correlation coefficient between the 2 observers is high, from 0.81 to 0.99 (p<10^-4^).

**Table 1:** Data as inter-observer reproducibility of measurements from 20 subjects

Measurement	Observer 1 Mean ± SD	Observer 2 Mean ± SD	*R*	*p* Value	Bland-Altman Mean ± SD
NC [g]	13.8 ± 3.3	11.7 ± 3.5	0.88	<10^-5^	-2.11 ± 1.68

C [g]	104.6 ± 21	103.7 ± 21	0.98	<10^-5^	-0.93 ± 4.48

NC/BSA [g/m^2^]	7.8 ± 1.7	6.6 ± 1.6	0.84	<10^-5^	-1.23 ± 0.95

C/BSA [g/m^2^]	58.9 ± 8.4	58.4 ± 8.8	0.96	<10^-5^	-0.46 ± 2.48

EDV [mL/ m^2^]	73 ± 15	72 ± 15	0.99	<10^-5^	-0.65 ± 1.12

Ratio NC/C [%]	13 ± 3	11 ± 3	0.81	<10^-4^	2.02 ± 1.7

## Conclusions

This semi-automatic method is highly reproducible and can be used to help diagnosis in LVNC patient and probably in first degree relative of patient with hypertrophic cardiomyopathy. Our first results show that the non-compacted mass in normal subjects is widely less than 10 g/m2 and the ratio between non compacted and compacted mass is always less than 15 %.

## Funding

N/A.

